# Prevalence and predictors of shared decision-making in goals-of-care clinician-family meetings for critically ill neurologic patients: a multi-center mixed-methods study

**DOI:** 10.1186/s13054-023-04693-2

**Published:** 2023-10-21

**Authors:** Victoria Fleming, Abhinav Prasad, Connie Ge, Sybil Crawford, Shazeb Meraj, Catherine L. Hough, Bernard Lo, Shannon S. Carson, Jay Steingrub, Douglas B. White, Susanne Muehlschlegel

**Affiliations:** 1 Departments of Neurology, University of Massachusetts Chan Medical School, Worcester, MA USA; 2 Tan Chingfen University of Massachusetts Graduate School of Nursing, Worcester, MA USA; 3 Department of Medicine, Oregon Health & Science University, Portland, OR USA; 4 Department of Medicine, University of California San Francisco, San Francisco, CA USA; 5 Division of Pulmonary and Critical Care Medicine, Department of Medicine, University of North Carolina Hospitals, Chapel Hill, NC USA; 6 Division of Pulmonary Medicine and Critical Care Medicine, Department of Internal Medicine, University of Massachusetts Medical School - Baystate, Springfield, MA USA; 7 Department of Critical Care Medicine, University of Pittsburgh School of Medicine, Pittsburgh, PA USA; 8 Departments of Anesthesia/Critical Care, University of Massachusetts Chan Medical School, Worcester, MA USA; 9 Departments of Surgery, University of Massachusetts Chan Medical School, Worcester, MA USA; 10 Departments of Neurology and Anesthesiology/Critical Care Medicine, Johns Hopkins University School of Medicine, 600 N. Wolfe St., Phipps 455, Baltimore, MD 21287 USA

**Keywords:** Shared decision-making, Neurocritical care, Clinician-family communication, Goals-of-care, Family meetings, End-of-life, Palliative care, End-of-life care, Stroke, Traumatic brain injury, SABI, Intracerebral Hemorrhage

## Abstract

**Background:**

Shared decision-making is a joint process where patients, or their surrogates, and clinicians make health choices based on evidence and preferences. We aimed to determine the extent and predictors of shared decision-making for goals-of-care discussions for critically ill neurological patients, which is crucial for patient-goal-concordant care but currently unknown.

**Methods:**

We analyzed 72 audio-recorded routine clinician-family meetings during which goals-of-care were discussed from seven US hospitals. These occurred for 67 patients with 72 surrogates and 29 clinicians; one hospital provided 49/72 (68%) of the recordings. Using a previously validated 10-element shared decision-making instrument, we quantified the extent of shared decision-making in each meeting. We measured clinicians’ and surrogates’ characteristics and prognostic estimates for the patient’s hospital survival and 6-month independent function using post-meeting questionnaires. We calculated clinician-family prognostic discordance, defined as ≥ 20% absolute difference between the clinician’s and surrogate’s estimates. We applied mixed-effects regression to identify independent associations with greater shared decision-making.

**Results:**

The median shared decision-making score was 7 (IQR 5–8). Only 6% of meetings contained all 10 shared decision-making elements. The most common elements were “*discussing uncertainty”*(89%) and *“assessing family understanding”*(86%); least frequent elements were *“assessing the need for input from others”*(36%) and *“eliciting the context of the decision”*(33%). Clinician-family prognostic discordance was present in 60% for hospital survival and 45% for 6-month independent function. Univariate analyses indicated associations between greater shared decision-making and younger clinician age, fewer years in practice, specialty (medical-surgical critical care > internal medicine > neurocritical care > other > trauma surgery), and higher clinician-family prognostic discordance for hospital survival. After adjustment, only higher clinician-family prognostic discordance for hospital survival remained independently associated with greater shared decision-making (*p* = 0.029).

**Conclusion:**

Fewer than 1 in 10 goals-of-care clinician-family meetings for critically ill neurological patients contained all shared decision-making elements. Our findings highlight gaps in shared decision-making. Interventions promoting shared decision-making for high-stakes decisions in these patients may increase patient-value congruent care; future studies should also examine whether they will affect decision quality and surrogates’ health outcomes.

**Supplementary Information:**

The online version contains supplementary material available at 10.1186/s13054-023-04693-2.

## Background

Annually, over 1 million Americans suffer a neurological emergency that renders them critically ill, either from a large ischemic or hemorrhagic stroke, traumatic brain injury, or other devastating neurological injury [1–3]. For critically ill neurological patients (CINPs), survival after injury often requires airway protection, artificial feeding support, and assistance for even the most basic needs. CINPs may be dependent on such high-level supportive care for weeks to months, or even years during a long-term and uncertain chronic recovery period [4]. Due to the incapacitating nature of severe neurological injury, CINPs also require a surrogate decision-maker, most often a family member, to make high-stakes decisions on continuation of life-sustaining treatments with an uncertain recovery and quality of life versus transition to comfort care with withdrawal of life-sustaining treatments (WLST), ultimately resulting in the death of the patient [5]. Many surrogates of CINPs are ill prepared for this “goals-of-care” decision-making process, resulting in significant psychological distress, poorly informed decisions, or even decision paralysis [6–8].

For high-stakes decisions, experts and professional critical care societies recommend shared decision-making (SDM) as “best practice” to achieve patient value-congruent and well-informed decisions, and to reduce decisional conflict, decision regret, and potentially surrogate psychological distress [9, 10]. The SDM model as developed by Charles et al. consists of three steps: information sharing, deliberation, and decision-making [10, 11]. In ICU contexts involving life support decisions and goals-of-care, physicians offer prognostication and treatment options, which the surrogate balances with their knowledge of the patient's values and preferences [5, 9, 10]. This collaborative approach to decision-making differs from both paternalism, where only the physician decides, and informed choice, where only the surrogate decides after receiving this information. SDM merges the physician’s medical expertise with the family's understanding of the patient's preferences. SDM is increasing in many parts of the world with some international variability [12–16], partly because of a recognition of the ethical imperative to properly involve patients in decisions about their care and partly because of the growing evidence that the approach has benefits [13].

In the critical care context, however, previous research in medical-surgical ICUs has shown that, despite efforts to implement SDM in family conferences, comprehensive SDM in goals-of-care decisions is generally incomplete [17]. Tools to facilitate comprehensive SDM (decision aids and other decision support interventions) have been developed for many other diseases and decisions and have been shown to improve knowledge sharing, reduce decisional conflict, and encourage active participation in the decision-making process [18, 19] Recently, one decision aid was developed for non-critically ill stroke patients [20]. A second decision aid has also been developed for critically ill patients with traumatic brain injury, hemorrhagic or large acute ischemic strokes [21, 22] with established feasibility of use [23]. Currently, there is no empirical data on SDM during goals-of-care discussions for CINPs before the broad implementation of decision aids. These data could encourage their implementation in routine practice and track their impact. The objective of this study was to describe the extent of SDM during goal-of-care clinician-family meetings for CINPs and to explore factors that may predict greater SDM.

## Methods

### Study design and enrollment

**Fig. 1:**
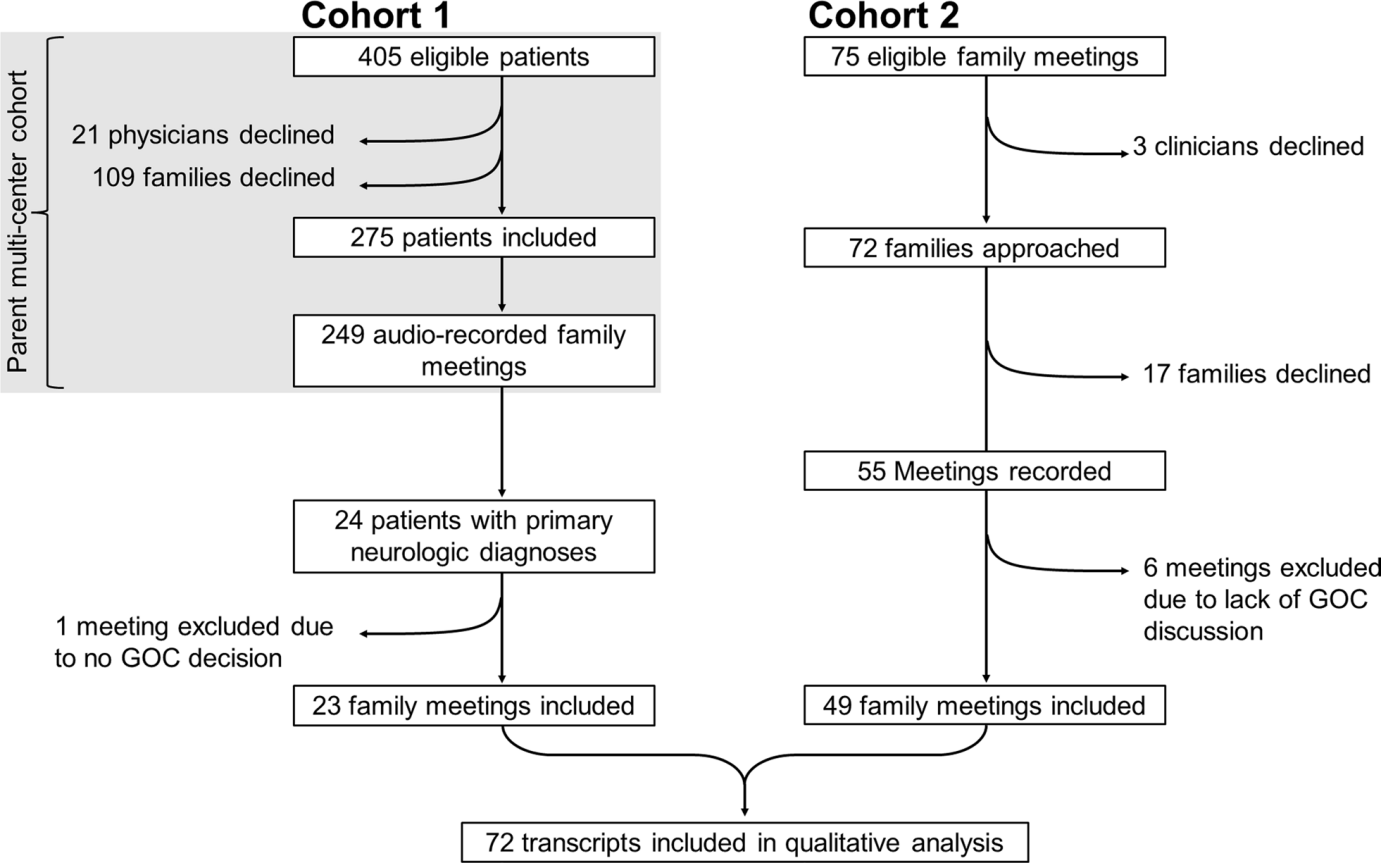
Study Flowchart. The data were pooled from two study cohorts. Cohort 1 included family meetings conducted in the medical-surgical ICU between 2009 and 2014 at six sites, with meetings specific to patients with primary neurologic diagnoses analyzed for this study. Cohort 2 consisted of family meetings conducted in a neurological ICU at one site between 2019 and 2021. We pooled and analyzed both cohorts together

We conducted a mixed-methods study of audio-recorded clinician-family meetings for CINPs. Study staff were informed by ICU staff about upcoming meetings deemed to be “goals-of-care” discussions by clinicians. Study staff then approached clinicians and surrogates for participation consent prior to audio-recording meetings. Meetings were conducted according to the local hospital’s standard of care. No decision aid or other intervention was used during the family meetings to facilitate the goals-of-care discussion. We excluded family meetings from analysis wherein prognosis or goals-of-care were not discussed (e.g., brief "update" about the current medical plan). We conducted a pooled analysis of clinician-family meeting transcripts, combining data from two cohorts (Fig. 1), with Institutional Review Boards approvals at the University of Massachusetts Chan Medical School (#H00016916) and University of Pittsburgh (#PRO09050285).

**Fig. 2:**
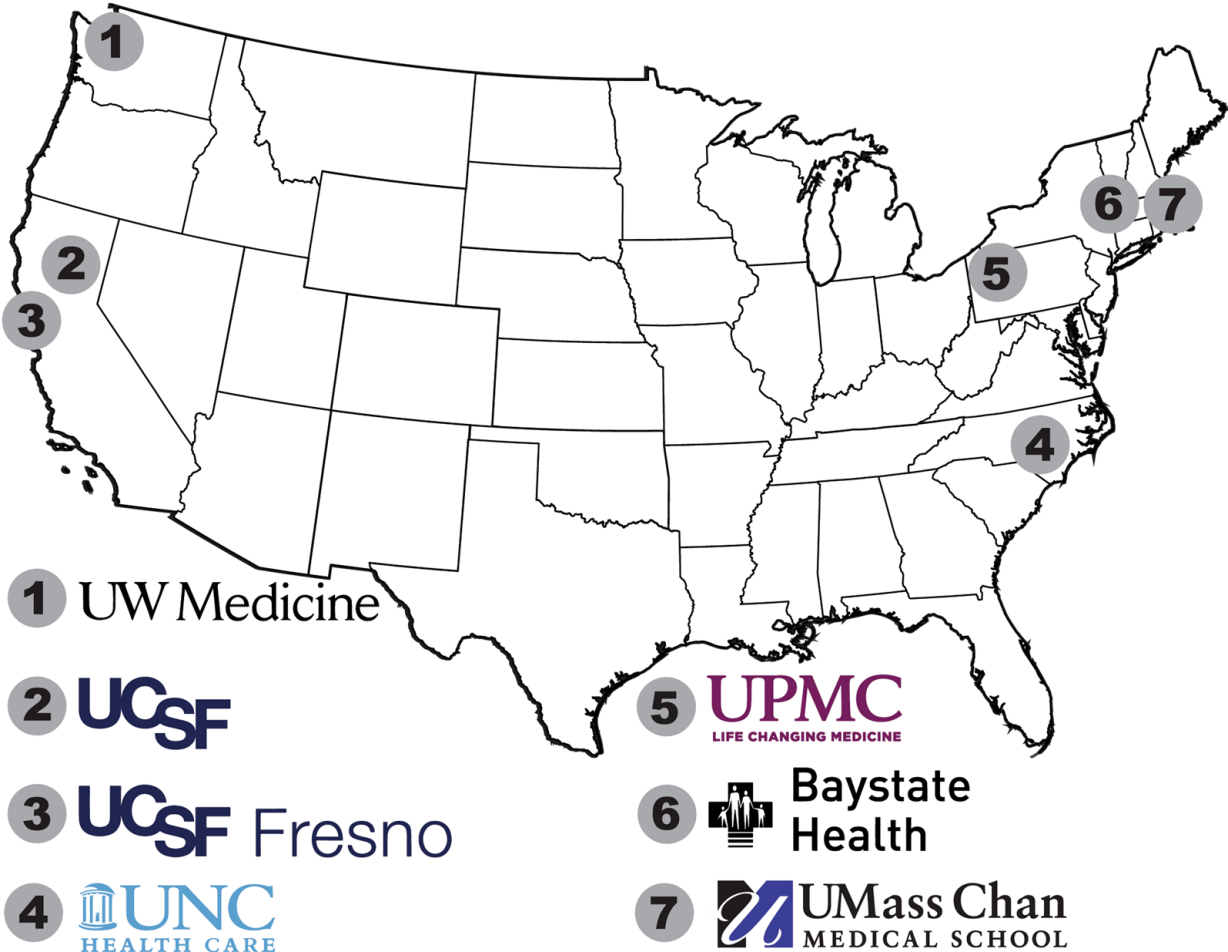
Study sites. Cohort 1 comprises meetings from medical-surgical ICUs at sites 1–6, while cohort 2 includes meetings from a neuroICU at site 7

Cohort 1 included a subset of clinician-family meeting recordings restricted to CINPs from a parent multicenter study implemented across 6 centers **(**Fig. 2**)**, which were recorded between 2009 and 2014. A full description of study methods has been previously published [24]. Briefly, surrogates were included for patients who were: ≥ 18 years old, medically incapacitated, in respiratory failure and mechanically ventilated, and had a calculated Acute Physiology Assessment and Chronic Health Evaluation II score of ≥ 25, predicting 40% chance of long-term severe functional impairment. Exclusion criteria were non-English-speaking surrogate decision-makers and patients on a waiting list for organ transplantation. All study participants provided written consent, and both surrogate and clinician participants received financial compensation for their time ($10–20). For this current study, only clinician-family meetings for patients with a primary neurological diagnosis were included.

Cohort 2 included clinician-family meetings recorded at the neurological ICU at the University of Massachusetts Chan Medical School/UMass Memorial Medical Center between 2019 and 2021. Study participants were recruited using an IRB-approved verbal recruitment script, provided verbal consent after reviewing an IRB-approved fact sheet, and did not receive financial compensation. Patient inclusion criteria were: ≥ 18 years old, incapacitated after a neurological emergency requiring ICU admission, and presence of a surrogate decision-maker ≥ 18 years.

After all clinician-family meetings, the primary decision-making surrogate and the clinician conducting the meeting completed questionnaires collecting demographic characteristics and prognostic estimates for the patient’s survival of hospitalization and return to independence at 6-months. Patient demographics and clinical data were abstracted from the medical record. The audio-recorded clinician-family meetings from both cohorts were professionally transcribed and de-identified prior to analysis. Transcripts were imported and coded with NVivo qualitative data analysis software (QSR International Pty Ltd. Version 12, 2018).

### Qualitative analysis

Three coders conducted the qualitative analysis. Two coders (A.P. and C.G.) developed an initial codebook on 5 recordings, by using a deductive approach by applying a previously validated 10-element SDM instrument [17], which encompasses the fundamental components of SDM as conceptualized by Charles et al., outlined in Table 1 [10, 11]. This deductive approach is a qualitative research method during which predetermined codes are applied to the data[25]. Additional file 1: Table S1 shows representative quotes for each SDM element. The transcripts were analyzed according to the presence or absence of the 10 elements of SDM, with the codebook including definitions and examples for each SDM element [17]. An element was considered present regardless of whether it was brought up by the clinician, or another member of the interdisciplinary care team, or family member. For example, Element 6, “*Elicit patient values and preferences”* was coded as present with statements by clinicians deliberately asking, “Had your loved one spoken to you about what they would like you to do in this situation?” to family members spontaneously and briefly exclaiming, “He wouldn’t want this!” A third coder (V.F.) was later trained to apply the established codebook to transcripts. Interrater reliability among all three coders was calculated on 7 of the same randomly selected transcripts, which were triple coded in parallel by all three coders. The inter-rater reliability (κ-statistic) was 0.86, indicating excellent interrater reliability. Coding discrepancies were reviewed and discussed with one senior investigator (S.M.) over a series of meetings and analysis of 5 additional transcripts. A total of 60 transcripts were then independently coded (V.F. 35, A.P. 25, C.G. 15) for a total of 72 analyzed transcripts.

**Table 1:** Validated 10-element shared decision-making instrument[17]

Dimensions of conversation	Elements of shared decision-making
Providing medical information	(1) Discuss the nature of the decision
	(2) Describe treatment alternatives
	(3) Discuss the pros and cons of the choices
	(4) Discuss uncertainty
	(5) Assess family understanding
Eliciting patient values and preferences	(6) Elicit patient values and preferences
Exploring the family’s preferred role in decision-making	(7) Discuss the family’s role in decision-making
Deliberation and decision-making	(8) Assess the need for input from others
	(9) Explore the context of the decision
	(10) Elicit the family’s opinion about the treatment decision

### Quantitative analysis

**Fig. 3:**
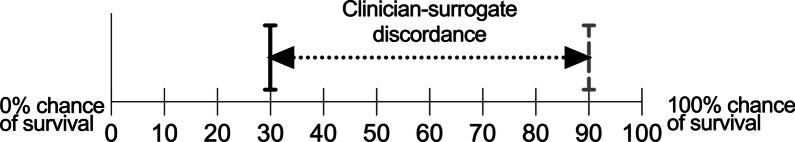
Calculation of the clinician-surrogate prognostic discordance. In the post-meeting questionnaires, clinician and surrogate respondents independently recorded their prognostic estimates of the patient’s chances of hospital survival and return to independence 6 months later on a horizontal probability scale (0–100%), anchored on the right and left. These anchors were intended to aid subjects with limited numeracy. We calculated the clinician-surrogate prognostic discordance by subtracting the clinician’s prognostic estimate from the surrogate’s estimate. Clinician-surrogate discordance was defined as clinician-surrogate differences of at least 20% using the absolute clinician-surrogate discordance value. In the example shown, the surrogate estimated that the patient has a 90% chance of hospital survival (gray striped line), and the clinician estimated a 30% chance (black line); hence, the discordance score is 60. Since we utilized absolute values, the discordance score remained the same regardless of whether the clinician's estimate was more optimistic (greater) or less optimistic (smaller) than the surrogate's

We summarized participant demographics using standard descriptive statistics. We calculated the SDM score for each clinician-family meeting by adding the number of SDM elements present in the meeting. This SDM score ranged from 0 (no SDM elements) to 10 (all 10 SDM elements present). We calculated the median SDM score (interquartile range [IQR]), mean (± standard deviation [SD]) and the frequency of each individual SDM element across all meetings. We calculated clinician-surrogate prognostic discordance by subtracting the clinician’s prognostic estimate from the surrogate’s estimate, separately for patient survival of hospitalization and 6-month return to independent function (Fig. 3). We defined clinician-surrogate prognostic discordance as the absolute difference of ≥ 20% between the clinician and surrogate estimate, which has previously been validated for assessing prognostic discordance between physicians and families of patients with severe acute brain injury [26]. The cut-off was determined based on a modified time-trade-off experiment in patients with serious illness, where patients' willingness to receive ongoing life-support declined substantially with a 20% worsening in prognosis [24, 27].

Unadjusted associations of patient, surrogate, and clinician characteristics with the SDM score were estimated using linear mixed modeling [28] with a random effect for center to account for within-center clustering. Multivariable analyses included variables with p-value < 0.2 in unadjusted analyses. The main analysis was a complete case analysis. In sensitivity analyses, we repeated the multivariable analyses after conducting multiple imputation of missing prognostic discordance (*n* = 20 for patient survival of hospitalization; *n* = 18 for 6-month return to independent function) using 5 rounds of sequential regression imputation [29]. Further details regarding the statistical analysis are listed in the Additional file 2. All analyses were conducted in SAS Version 9.4 (SAS Institute, Inc., Cary, North Carolina, U.S.). PROC MIXED was used to estimate the linear mixed models for SDM score.

## Results

Across both cohorts, we included a total of 72 clinician-family meetings for 67 CINPs with 72 surrogates and 29 clinicians from 7 sites. From cohort 1, we included 23 clinician-family recordings from 23 patients with a primary neurologic diagnosis from an original data set of n = 275 meetings. From cohort 2, we included 49 clinician-family recordings for 44 patients. Of these 49 meetings, 12 had partially incomplete post-meeting questionnaires. The average duration of the audiorecorded meetings was 36 ± 17 min. Clinician-family meetings occurred at an average 7.5 ± 6.6 days after ICU admission.

Characteristics for all participants are shown in Table 2. Nine (31%) of the 29 participating clinicians were female; because some clinicians participated in more than one meeting, 54% of recorded meetings were led by a female clinician. Clinician specialties varied but were most commonly in internal medicine (28%), medical/surgical critical care (21%) and neurocritical care (21%). In cohort 1, most were in internal medicine (39%) or critical care (30%), whereas in cohort 2 most clinicians were in neurocritical care (60%). In both cohorts combined, most meetings were led by an attending physician (76%), vs. resident, fellow, or advanced practice provider (24%). Cohort 2 featured a higher percentage of attending-led meetings (88%) as compared to cohort 1 (52%). The majority of patients (70%), surrogates (67%), and clinicians (82%) identified as non-Hispanic White. Among patients in the entire cohort, admission diagnoses were most commonly acute ischemic stroke (33%) and traumatic brain injury (24%). Health literacy and education levels were very high among all participating surrogates; surrogates scored an average of 6.9 out of 7 on the REALM estimate of health literacy [30] and an average of 7 out of 11 on the numeracy scale [31] (for both scales higher numbers indicate higher levels of health literacy or numeracy). Of the surrogates who filled out the post-meeting questionnaire, 70% reported attained at least “some college” as their highest level of education, and 40% earned a college or graduate-level degree.

**Table 2:** Participant characteristics of patients, family members, and clinicians

Characteristic (*n* [%], unless otherwise noted)	Patients (*n* = 67)	Surrogates (*n* = 72)	Clinicians (*n* = 29)
*Age* (years), mean (SD)	62 (19)	48 (13)	43 (8)
*Sex*			
Male	31 (46%)	26 (36%)	20 (69%)
Female	29 (43%)	33 (46%)	9 (31%)
Declined to answer	0	13 (18%)	0
*Race/ethnicity*			
Non-Hispanic White	47 (70%)	48 (67%)	22 (76%)
Hispanic White	6 (9%)	2 (3%)	2 (7%)
Asian	4 (6%)	4 (6%)	3 (10%)
Black	2 (3%)	4 (6%)	2 (7%)
Native American/Alaska Native	0	1 (1%)	0
Declined to answer	0	13 (18%)	0
*Diagnoses*			
Traumatic Brain Injury	16 (24%)		
Acute Ischemic Stroke	22 (33%)		
Aneurysmal Subarachnoid Hemorrhage	6 (9%)		
Hemorrhagic Stroke	11 (16%)		
Other			
(Encephalitis, status epilepticus, neoplasm, hypoxic ischemic brain injury)	12 (18%)		
*Highest level of education*			
Less than High school		3 (4%)	
High school graduate or GED		13 (18%)	
Some college		11 (15%)	
2 years college or technical school		5 (7%)	
College graduate		15 (21%)	
Graduate school or professional degree		11 (15%)	
Declined to say		14 (19%)	
*Measures of literacy and numeracy, median (IQR)*			
REALM Estimate of Health Literacy		7 (7;7)	
General Numeracy Scale Score		7 (5.75;9)	
*Practice level*			
Attending physician			16 (55%)
Resident, fellow, APP			13 (45%)
*Years of practice*			*14 (8%)*
*Clinician specialty*			
Neurocritical Care			6 (21%)
Med/Surg Critical Care			6 (21%)
Internal Medicine			8 (28%)
Trauma Surgery			1 (3%)
Other (Nephrology, neurosurgery, general surgery, palliative care, anesthesia)			8 (28%)

### Prevalence of shared decision-making

**Fig. 4:**
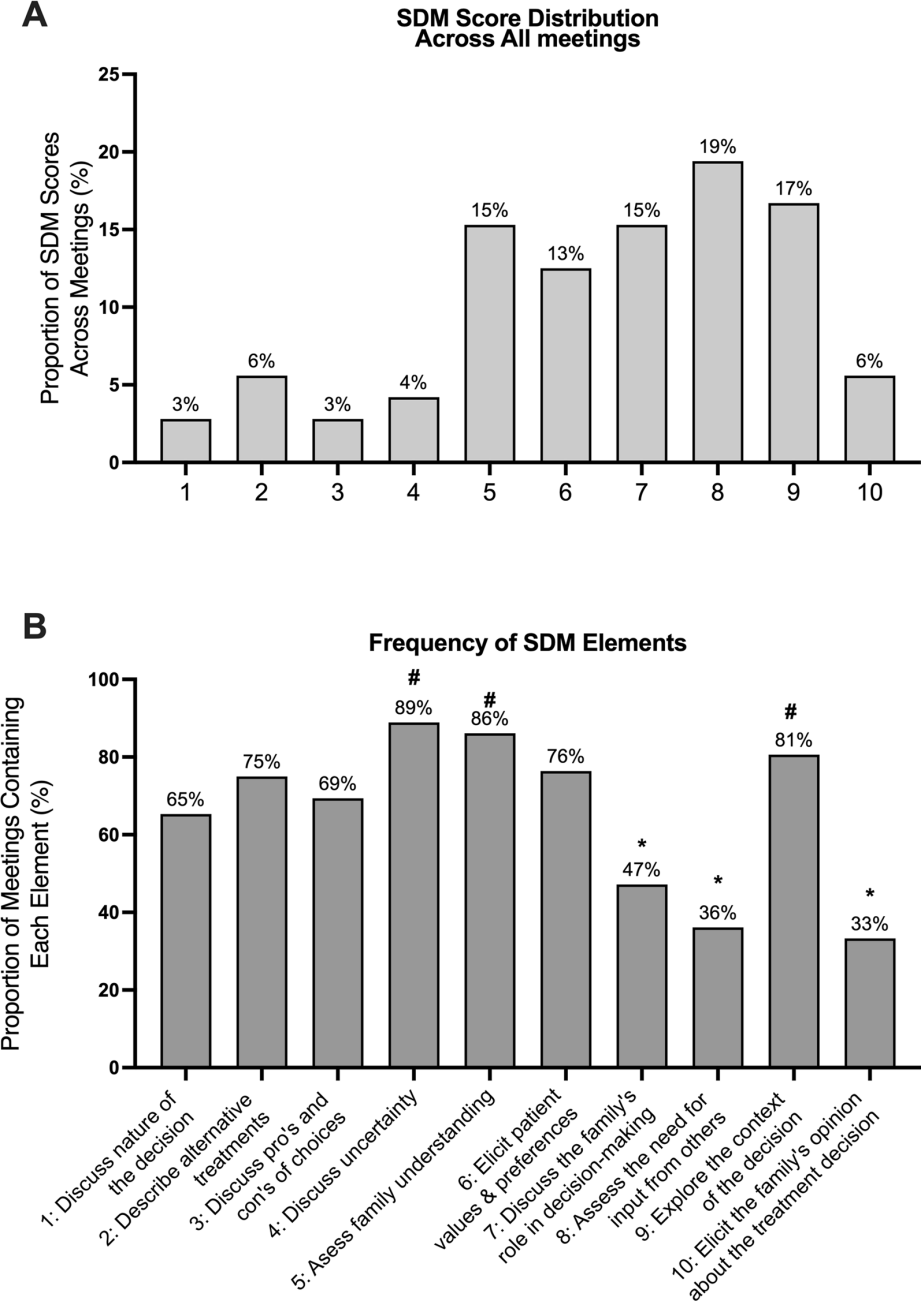
Panel **A** shows the proportion of SDM scores across all meetings. SDM scores can range from 0 (lowest) to 10 (highest). There were no meetings with an SDM score of 0. Panel **B** displays the frequency of SDM elements, summarized as the proportion of meetings containing each of the 10 SDM elements. Hash (#) indicates the three most frequent and asterix (*) the three least frequent elements across all meetings

The median SDM score was 7 (IQR 5–8), and the mean (± SD) SDM score was 6.5 ± 2.3. Only 4 (6%) meetings contained all 10 elements of SDM (Fig. 4A). There was substantial variation in the frequency of SDM elements (Fig. 4B). The most prevalent SDM element was #4, “*Discussing uncertainty”* (89% of meetings); the second-most prevalent element was #5, “*Assessing family understanding”* (86%); and the third-most prevalent element was #9 “*Exploring the context of the decision*” (81%). Fewer than half of the meetings contained elements #7, “*Discuss the family’s role in decision-making”* (47%)*, #* 8, “*Assess the need for input from others”* (36%), and #10, “*Elicit the family’s opinion about the treatment decision”* (33%). Additional file 2: Figures S1A-C and Table S2 reveal a more detailed subgroup analysis of the SDM Score (see Additional File 2); Additional Fig. 2 shows the distribution of the SDM score by center (see Additional file 2).

### Associations with greater SDM

Eighty-two percent (59/72) of family members and 93% (67/72) of clinicians completed the post-meeting questionnaire about demographics, health literacy, numeracy, perception of conflict with the family, and prognostic estimates. Table 3 shows the univariate associations with the total SDM score. Lower clinician age (*p* = 0.012) and fewer clinician years in practice (*p* = 0.019) were both significantly associated with greater SDM. Clinician specialty was also associated with greater SDM (*p* = 0.034), with SDM being highest among medical/surgical intensivists, followed by internal medicine clinicians and neurointensivists, and lowest among trauma surgery providers. SDM also was significantly higher in meetings with clinician-surrogate prognostic discordance regarding hospital survival (*p* = 0.028). Additional file 1: Figures S2 and S3 show scatterplots of SDM vs. survival discordance by center and cohort. None of the other variables were significantly associated with SDM in univariate analysis. However, patient diagnosis (*p* = 0.188) and clinician-surrogate prognostic discordance about 6-month independence (*p* = 0.156) crossed the threshold to be included in the multivariable model (unadjusted *p* < 0.20).

**Table 3:** Univariate associations of SDM score with patient, surrogate, and clinician characteristics (adjusted for random center effect, except where noted)

Characteristic	Mean (Std Error) or *β* (Std Error)	*p* value
*Patient*		
Age in years	− 0.005 (0.02)	0.763
Sex		0.472
Female	6.06 (0.59)	
Male	6.44 (0.57)	
Race/ethnicity		0.409
Non-Hispanic White	6.15 (0.49)	
Hispanic	7.55 (1.01)	
Asian	7.09 (0.98)	
Black	6.12 (1.62)	
Diagnosis		0.188
Traumatic brain injury	5.63 (0.57)	
Acute ischemic stroke	6.77 (0.58)	
Aneurysmal subarachnoid hemorrhage	6.47 (0.77)	
Intracerebral hemorrhage	7.69 (0.79)	
Other	5.89 (0.93)	
*Surrogate*		
Education		0.728
≤ high school	6.16 (0.69)	
Any college	6.16 (0.63)	
Post-college	6.79 (0.84)	
Conflict with clinical team ^(a)^		0.312
1	6.31 (0.39)	
2	7.35 (0.56)	
3–4	6.33 (1.33)	
Trust in clinical team		0.759
1	6.73 (0.84)	
2	5.94 (1.20)	
3	6.18 (0.72)	
4	6.88 (0.76)	
*Clinician*		
Age	− 0.08 (0.03)	0.012
Years in practice	− 0.07 (0.03)	0.019
Attending		0.841
No	6.36 (0.69)	
Yes	6.23 (0.56)	
Sex		0.566
Female	6.48 (0.61)	
Male	6.16 (0.53)	
Race/ethnicity		0.772
Non-Hispanic White	6.39 (0.49)	
Hispanic	6.42 (1.38)	
Asian	5.78 (0.87)	
Black	5.07 (1.67)	
Specialty		0.034
Neurocritical care	6.22 (0.78)	
Medical/surgery critical care	7.69 (0.82)	
Internal Medicine	6.29 (0.85)	
Trauma surgery	3.62 (1.62)	
Other	4.77 (0.76)	
Perception of conflict with family		0.925
1	6.15 (0.52)	
2	6.16 (0.78)	
3	6.74 (0.89)	
4	6.23 (1.38)	
Clinician-surrogate prognostic discordance		
Survival of hospitalization ^(b)^		0.028
Discordant	6.90 (0.61)	
Concordant	5.48 (0.66)	
6-month independence ^(c)^		0.156
Discordant	5.84 (0.54)	
Concordant	6.78 (0.49)	

^(a)^Not adjusted for random center effect, as estimated random effect variance was 0

^(b)^Missing for 20 meetings

^(c)^Missing for 18 meetings

Table 4 shows the results of multivariable analysis. Clinician years in practice was highly collinear with age (Pearson *r* = 0.90) and, thus, was not included. The Pearson correlation between the two discordance variables was low, at 0.20 (Spearman correlation = 0.15), indicating no collinearity. After adjustment, clinician-surrogate prognostic discordance regarding hospital survival remained as an independent predictor of greater SDM (mean [standard error] = 7.00 [0.40] versus 5.59 [0.46] for concordance; *p* = 0.029). Results from the sensitivity analyses after multiple imputation were consistent with the main analysis, except that clinician-surrogate prognostic discordance regarding hospital survival was no longer independently associated with SDM (*p* = 0.296).

**Table 4:** Multivariable analysis of factors associated with higher SDM. (main analysis with complete case analysis)

Characteristic	Mean (Std Error) or β (Std Error)	*p* value
*Patient*		
Diagnosis		0.379
Traumatic brain injury	5.54 (0.63)	
Acute ischemic stroke	6.77 (0.53)	
ASH	5.73 (0.90)	
Hemorrhagic stroke	7.69 (0.86)	
Other	6.51 (1.143)	
*Clinician*		
Age^(a)^	− 0.0648 (0.0520)	0.22
Specialty		0.072
Neurocritical care	6.42 (0.42)	
Medical/surgery	7.96 (1.00)	
Internal Medicine	6.17 (0.79)	
Trauma surgery	7.42 (1.89)	
Other	3.15 (1.31)	
Discordance, hospitalization survival		0.029*
Discordant	7.00 (0.40)	
Concordant	5.59 (0.46)	
Discordance, 6-month independence		0.423
Discordant	6.10 (0.48)	
Concordant	6.65 (0.42)	

^(a)^Pearson correlation of clinician age with years in practice is 0.90; including both as variables would yield collinearity, thus only age is included in the model

*denotes statistical significance in multivariable model (*p* < 0.05)

## Discussion

Our study demonstrated that SDM utilization is limited in goals-of-care clinician-family meetings for CINPs and that complete SDM is rare. Only 6% of family meetings contained all 10 elements of SDM. Our study also uncovered specific gaps in SDM use, as clinicians often failed to inquire the role the family preferred to take as a decision-maker and about consulting other stakeholders who might not be present in the meeting.

Our results partially mirrored those from prior research of audio-recorded clinician-family meetings in medical-surgical ICUs, which demonstrated frequent shortcomings of SDM [17, 32]. For example, in one study using the same methodology as in our study, only a minority (2%) of family meetings included all components of SDM [17]. In that study, like ours, clinicians also failed to explore the family’s role in decision-making or assess for the need for input from others in most meetings. Conversely, in this previous study, clinicians rarely assessed family understanding, while clinicians in our study assessed family understanding in over 80% of meetings. In addition, clinicians in our study discussed both prognostic uncertainty and the pros and cons of the medical decision at higher rates (89% and 69%, respectively). One possible explanation may be differences in the patient populations being studied: only a minority of patients from the prior study had a primary neurological disorder and were treated in specialized neuroICUs, where prognostic uncertainty is very common [33, 34]. An alternative explanation for the differences between this study and previous ones may arise from varying regional and situational SDM practices [35].

Conversations regarding goals-of-care and prognosis for CINPs should encompass a dialogue about uncertainty and functional outcomes with the potential for a prolonged rehabilitation course. Prior research has shown that clinicians find uncertainty frustrating and that they especially worry that their prognostication may create false hope in the presence of uncertainty [33, 34]. In a recent study of prognostic uncertainty for TBI patients, most clinicians found uncertainty to be present during prognostication and most compensated for that by describing many possible outcomes [33]. It is possible that the high uncertainty of recovery following neurological injury prompted clinicians in our study to similarly “compensate” with increased discussions of uncertainty and more frequent assessments of family understanding.

Previous studies have shown that families generally want to be involved in the patient’s ICU care [6, 36]. These studies have shown that many family members who acted as surrogate decision-makers and observed their loved one go through an ICU hospitalization expressed a wish for greater participation and involvement in the treatment decision-making process; they preferred shared decision-making over the burden of individual decision-making, which was found to be a source of anxiety [6, 36]. Seeking input from the patient's loved ones, such as family members, friends, and clergy, is vital to establish trust with the family, gain a comprehensive understanding of the patient as an individual, and thoroughly examine their values and preferences from various perspectives [6, 37]. This is particularly important in neurological emergencies, which can occur without warning and may result in life-long disability.

Nonetheless, surrogates may have varying preferences about the decision-making role they would like take [38]; yet, clinicians in our study asked about that preferred role in less than half of meetings. This is similar to a prior study in medical-surgical ICUs, where clinicians also commonly avoided exploring the role of the surrogate decision-maker [17]. These findings have significant implications, as some family members may find themselves taking on a more substantial role in medical decision-making than they would like, leading to elevated rates of anxiety and depression [39]. In addition, the preferred degree of involvement of a surrogate may shift during the patient's hospitalization as they become more familiar and accustomed to the ICU treatment plan [6, 9]. For these reasons, we propose that clinicians consistently ask about the surrogate’s desired role for decision-making at different time points during the patient’s ICU stay.

In our study, meetings with higher levels of clinician-surrogate prognostic discordance surrounding the patient's hospital survival were associated with greater SDM. In contrast, prior research has proposed a connection between greater prognostic discordance and ineffective communication by clinicians, such as the failure to verify family comprehension of the clinical situation [26]. The reasons for this association are unclear. One possible explanation that some have proposed is that this kind of discordance may serve as a type of “performative optimism”, whereby family members maintain an optimistic outlook in the hope of ultimately improving the patient's clinical outcome [24]. Prior studies have found that prognostic discordance arises not from lack of medical information but rather from psychosocial factors such as religious belief, performative optimism, and cognitive bias [24]. In our study, clinicians may have intuited prognostic discordance during their discussions with family members during the family meeting leading them to employ more SDM in an attempt to close a perceived knowledge gap or psychosocial distress. Alternatively, our study may have revealed the surrogates' inclination to over- or underestimate prognostic information, as higher SDM in meetings may reflect the inclusion of more information overall [40].

Our sensitivity analysis after multiple imputation no longer revealed an independent association between prognostic discordance regarding patient hospital survival and higher SDM. This may be explained by the introduction of uncertainty and variability through multiple imputations, which can affect the estimated associations between variables [41]. The imputed values may not perfectly represent the true values, and the imputation model may not fully capture the underlying relationships, which can lead to differences in the results compared to our complete case analysis.

Although prior research suggests that greater SDM occurs with more educated surrogates [17], we did not confirm this in our study. The overall high education levels of the surrogates in our study may account for this finding, as they may have reduced the regression model's sensitivity to detect an association between education level and SDM scores.

This study has several important strengths. This study merged two cohorts to incorporate audio-recordings of clinician-family meetings on goals-of-care from seven medical centers in the USA, thereby enhancing geographic diversity and generalizability. The clinicians involved in our study came from different specialties and had varying levels of experience. Capturing goals-of-care clinician-family meetings, which often entail end-of-life decisions, is a challenging task due to the significant effort and sensitivity required to recruit and retain participants. Thus, our cohort of 72 audio-recorded meetings for critically ill neurological patients is relatively large. We used a validated framework in our analyses. Although we had limited control over variables compared to simulation-based studies, our empirical research describes real-world clinical practice and may provide practical suggestions for improving clinician-family meetings.

This study has several notable limitations. Limited racial and ethnic diversity restricts the generalizability of our findings. The clinicians' knowledge of their participation in the study may have influenced their communication with families during these meetings (Hawthorne Effect) [42], potentially skewing the recordings toward more comfortable and less conflicted conversations. Additionally, since the audio-recorded and transcribed family meetings were occasionally one of several held between the clinicians and families, we may have missed some meetings and additional elements of SDM used in non-recorded meetings. This study combined two cohorts and analyzed two datasets that were collected almost a decade apart, potentially leading to confounding and discrepancies within the datasets due to changes in SDM awareness and practice over time. We attempted to mitigate this by adjusting for "cohort" during our multivariable analysis. Other factors that could have impacted our regression model's results include collinearity among variables, a limited sample size, and restricted variability within certain categories. Our observational study did not collect data on religious beliefs in all included cohorts or capture detailed insights into families' psychosocial distress or satisfaction with SDM post-meeting. Recruiting surrogate participants for end-of-life care and decision-making studies can be challenging, as they are often distressed and grieving. Families may be even more overwhelmed after a clinician-family meeting that delivers a guarded or poor prognosis, resulting in some surrogates declining to complete post-meeting questionnaires and incomplete data for our quantitative analysis. Decision-making authority varies between different countries and cultures, but this does not affect the findings of this empirical study. Rather than focusing on decisional authority, we centered on information exchange, grasping patients' values and preferences, and other globally relevant principles.

## Conclusions

Our study provides empirical data on the current prevalence and gaps of SDM in CINPs. Interventions promoting shared decision-making for high-stakes decisions in these patients may increase patient-value congruent care; future studies should also examine whether they will affect decision quality and surrogates’ health outcomes, and further explore prognostic discordance.

### Supplementary Information

**Additional file 1.** Supplementary Table 1.

**Additional file 2.** Additional details to the statistical analysis, SDM score subgroup analysis, Supplementray Table 2 and Figures S1–S4.

## Data Availability

Anonymized data not published within this article will be made available by request from any qualified investigator after signing a data use agreement.

## Abbreviations

SDM: Shared decision-making
ICUs: Intensive care units
CINPs: Critically ill neurological patients
WLST: Withdrawal of life-sustaining therapies
