# Supplementary material for: Prevalence and predictors of shared decision-making in goals-of-care clinician-family meetings for critically ill neurologic patients: a multi-center mixed-methods study
Source: Crit Care. 2023 Oct 21;27:403. doi: 10.1186/s13054-023-04693-2 (PMC10590503; doi:10.1186/s13054-023-04693-2)
Supplement: Supplementary file 1 — Additional file 1. Supplementary Table 1. [file 13054_2023_4693_MOESM1_ESM.docx]

**Additional File 1**

**Additional Table 1: Validated 10-Element Shared Decision-Making Instrument with representative quotes**

| **Dimensions of Conversation** | **Elements of Shared Decision-Making** | **Representative Quotes** |
| --- | --- | --- |
| Providing medical information | (1) Discuss the nature of the decision | MD: *“So the reason we wanted to meet with you is just number one, to give you an overview of what's going on and make sure that you understand everything, but also because there are some decisions that are now coming up, uh, and that have to be made for her, uh, so that we do, you know, provide the best care possible for her.”*  MD: *“The reason we’re here today is to talk about goals of care. That’s really what this decision is about. At this point, you have two options. Let me just explain the options to you."* |
|  | (2) Describe treatment alternatives | MD: *“OK, so I did mention -- I mentioned the option of goal is survival and try to get him to rehab and so forth. He has another option. The other option is not to focus on survival, but to focus on comfort. But it would also mean accepting that we're not going to put a feeding tube down, right? We're not going to...what we're doing right now is artificially prolonging his life.”*  MD: *“Option number one is to do everything, and the goal of care is survival no matter what it takes…Option number two is to not go with survival as the goal, but that comfort is the goal.”* |
|  | (3) Discuss the pros and cons of the choices | MD: *“So that’s a feeding tube …and the tracheostomy. I think both things will help with comfort right now. They may help her get off the machines. The problem with doing those things is that sometimes they’re not able to be reversed. And they change when you … where you can go when you leave the hospital. Okay? So people that have the tracheostomy and...if they’re not able to get off the machine right away, they have to go to a nursing facility, like a long-term care facility.”*  MD: *“He feels that that procedure is unlikely to make any difference, but he would be willing to do it to try, but he thinks it's going to be a risk for no benefit. So, he thinks that the procedure has some risk to it and there's some risk of infection, there are risks of damage to the brain...”* |
|  | (4) Discuss uncertainty | MD: *“To be completely honest with you, I do not know how much of that is permanently or reversibly damaged.”*  MD: *“But there’s a lot of grey area. Whether she’ll need just some assistance and be able to walk with a walker but by herself or whether she’ll not even be able to get out of the bed by herself. And it’s hard for me to put where on there … she’s going to go.”* |
|  | (5) Assess family understanding | MD: *“I'll stop here and ask you first, do you have any questions about what happened to her and what we're doing for her?”*  MD: *“I always like the family to talk and give me your impression, your understanding of what you’ve heard from others, what you understand. Kind of summarize to me what you’ve learned from the team, what’s going on.”* |
| Eliciting patient values and preferences | (6) Elicit patient values and preferences | MD: *“But in general, let's say he was sitting in the back of the room, what kind of quality of life is important for him? What is it that he would like to do, and what is it that he would accept as okay still, even if we... we told you that he may not return back to his baseline, which, in a stroke of this size, is very likely.”*  MD: *“I mean that's certainly a decision that you could make--if you decide together as a family, based on what [the patient] would want, that under no circumstances--temporary or with some improvement--would she want a feeding tube, then we will respect that decision.”* |
| Exploring the family’s preferred role in decision-making | (7) Discuss the family’s role in decision-making | MD: *“I will return that question to you because that's not a question really for the medical team to answer, that's more of a question for the family to answer, right?”*  MD: *“In his case, because he’s in the coma, it would mean that you as his family would decide, would give consent to do a tracheostomy.”* |
| Deliberation and decision-making | (8) Assess the need for input from others | MD: *“Do you want so, um, is there, are there others, you mentioned your daughter, who's making decisions. Do you want to FaceTime with her with mom there?”*  MD: *“And are you all the children, or are there more? All the decision makers together?”* |
|  | (9) Explore the context of the decision | MD: *“And so, with this stroke on top of that baseline, my prediction would be that he would never get out of bed again. And that he would be complete nursing care, completely dependent on being turned, being fed, needing a feeding tube, probably lifelong. Maybe, probably, most likely even needing a tracheostomy.”*  MD: *“My best guess would be a few hours. Yeah, maybe a day or two at best, but not substantial amounts of time. But the goal comfort is that she would pass away naturally with as much comfort as we can give her, and it's often pain medicines, medicines against anxiety, and making sure that she's not suffering, and that she's kind of peacefully able to pass. Essentially, let nature do what nature would do if we did not have machines.”* |
|  | (10) Elicit the family’s opinion about the treatment decision | MD: *“Are you okay with a no escalation of care?”*   Family: *“It's even if he were to get somewhere, it'd be such a long road that I don't know if he...would want it. 'Cause again he doesn't want to be -- I swear he knew that this was coming, and he just didn't say it. I mean, obviously he did, but the way that he said everything it was like, "OK, I don't want to be like this, and this is how it is,"...Yeah, we were driving down the road and it was like just a random conversation. “Hey if anything were to happen to me, just know, I don't want to live like a vegetable." And I'm like..."what?" He’s like, "I don't want to be resuscitated. I don't want to have something breathing for me. I don't want to walk around with tubes in me."* |
