# Supplementary material for: Prevalence and predictors of shared decision-making in goals-of-care clinician-family meetings for critically ill neurologic patients: a multi-center mixed-methods study
Source: Crit Care. 2023 Oct 21;27:403. doi: 10.1186/s13054-023-04693-2 (PMC10590503; doi:10.1186/s13054-023-04693-2)

**Additional file 2**

**Additional details to the statistical analysis**

We estimated bivariate (unadjusted) associations of each patient, surrogate, and clinician characteristic with SDM score, including a random effect for center to account for clustering [23]; further clustering within clinician was negligible (variance of the clinician random effect was 0). In sensitivity analyses, we re-ran models omitting meetings other than a patient’s first meeting, as there were only 3 such patients, too few to include a random patient effect; all results were similar (data not shown). In multivariate analyses, all variables with bivariate p<.20 (with the exception of clinician years of practice, which was highly correlated with clinician age; Pearson correlation=0.90) were included simultaneously as predictors, with and without forcing in patient diagnosis. The main analysis was a complete case analysis. In sensitivity analyses, we repeated the multivariable analyses after conducting multiple imputation of missing prognostic discordance (n=20 for patient survival of hospitalization; n=18 for 6-month return to independent function) using 5 rounds of sequential regression imputation[23].

**SDM score subgroup analysis**

*Comparisons Between First and Fourth SDM Score Quartiles*

The mean SDM score within the first SDM Score Distribution quartile was 3 (n=11) with scores ranging from 1-4. The mean SDM score within the fourth quartile was 9 (n=14) with scores ranging from 9-10. **Additional** **Table 2** summarizes the participant characteristics between the first and fourth SDM score distribution quartiles. Across both quartiles, there was an even split between male and female clinicians (45% male in the first quartile and 50% male in the second quartile). Race and ethnicity for participants largely mirrored the overall distribution of participants with the majority of patients (100% of patients in the first quartile, 79% in the second quartile), family members (91% in the first quartile and 57% in the fourth quartile), and clinicians (82% in the first quartile and 100% in the fourth quartile) identifying as non-Hispanic white. Family members in both quartiles were well educated with 64% of family members in the first quartile and 71% of family members in the fourth quartile having completed at least some college.

**Additional Table 2. Participant characteristics of patients, family members, and clinicians between first and fourth SDM score quartiles**

|  | **Fourth Quartile  Patients (n=14)** | |
| --- | --- | --- |
| **Age, mean (SD)** |  |  |
| Age (years) | 54 (23) | 62 (17) |
| **Sex, n (%)** |  |  |
| Male | 5 (45) | 7 (50) |
| Female | 6 (55) | 7 (50) |
| Declined to answer | 0 | 0 |
| **Race/ethnicity, n (%)** |  |  |
| Non-Hispanic White | 11 (100) | 11 (79) |
| Hispanic White | 0 | 2 |
| Asian | 0 | 1 |
| Black | 0 | 0 |
| Native American / Alaska Native | 0 | 0 |
| Declined to answer | 0 | 0 |
| **Diagnoses, n (%)** |  |  |
| Traumatic Brain Injury | 6 (55) | 4 (29) |
| Acute Ischemic Stroke | 2 (18) | 5 (36) |
| Aneurysmal Subarachnoid Hemorrhage | 1 (9) | 0 |
| Hemorrhagic Stroke | 1 (9) | 5 (36) |
| Other (Encephalitis, status epilepticus, neoplasm, hypoxic ischemic brain injury) | 1 (9) | 0 |
|  | **Fourth Quartile Surrogates (n=14)** | |
| **Age, mean (SD)** |  |  |
| Age (years) | 40 (15) | 50 |
| **Sex, n (%)** |  |  |
| Male | 2 (18) | 8 |
| Female | 9 (82) | 5 |
| Declined to answer | 0 | 1 |
| **Race/ethnicity, n (%)** |  |  |
| Non-Hispanic White | 10 (91) | 8 |
| Hispanic White | 0 | 2 |
| Asian | 0 | 2 |
| Black | 1 (9) | 1 |
| Native American / Alaska Native | 0 | 0 |
| Declined to answer | 0 | 1 |
| **Highest level of education, n (%)** |  |  |
| Less than High school | 0 | 1 |
| High school graduate or GED | 3 (27) | 2 |
| Some college | 3 (27) | 1 |
| 2 years college or technical school | 1 (9) | 3 |
| College graduate | 2 (18) | 4 |
| Graduate school or professional degree | 1 (9) | 2 |
| Declined to say | 1 (9) | 1 |
| **Measures of Literacy, median (IQR)** |  |  |
| REALM Estimate of Health Literacy | 7 (7-7) | 7 (7-7) |
| General Numeracy Scale Score | 4 (3-6) | 8 (8-10) |
|  | **Fourth Quartile Clinicians (n=14)** | |
| **Age, mean (SD)** |  |  |
| Age (years) | 47 (11) | 41 (4) |
| **Sex, n (%)** |  |  |
| Male | 5 (46) | 3 (21) |
| Female | 6 (54) | 11 (79) |
| **Race/ethnicity, n (%)** |  |  |
| Non-Hispanic White | 9 (82) | 14 |
| Hispanic White | 0 | 0 |
| Asian | 1 (9) | 0 |
| Black | 1 (9) | 0 |
| Native American / Alaska Native | 0 | 0 |
| Declined to answer | 0 | 0 |
| **Practice level, n (%)** |  |  |
| Attending physician | 7 (64) | 12 (86) |
| Resident/Fellow/APP | 4 (36) | 2 (14) |
| **Years of practice, mean (SD)** |  |  |
|  | 19 (12) | 13 (5) |
| **Clinician specialty, n (%)** |  |  |
| Neurocritical Care | 4 (36) | 11 (79) |
| Med/Surg Critical Care | 1 (9) | 2 (14) |
| Internal Medicine | 2 (18) | 0 |
| Trauma Surgery | 2 (18) | 0 |
| Other | 2 (18) | 1 (7) |

**Additional Figure 1A-C**

**Additional Figure 1A** shows the distribution of patient neurological diagnoses by SDM Score quartile. Notably, although fewer than a quarter (22%) of total meetings were held for patients with TBI, 54% of meetings in quartile 1 were for patients with TBI as compared with 29% in quartile 4. Additionally, while 58% of meetings in this study were run by neurocritical care specialists, 36% of meetings in quartile 1 and 79% of meetings in quartile 4 were run by neurocritical care specialists.

**Additional Figure 1B** shows the proportion of meetings led by clinicians from different specialties. The fourth quartile did not contain any meetings run by trauma surgeons or internal medicine specialists.

**Additional Figure 1C** shows the distribution of Numeric Literacy Scale scores among surrogates in the first and fourth SDM score quartiles. The dark gray bars represent the first quartile score distribution, and the light gray bars demonstrate the fourth quartile score distribution.

**Additional Figure 2: Distribution of the SDM score by center**

For each center, the bottom line of the box indicates the 25^th^ percentile, the middle line indicates the median (50^th^ percentile), and the top line indicates the 75^th^ percentile.  Error bars below and above the box indicate the 10^th^ and 90^th^ percentiles, respectively, and circles outside the error bars indicate outlying values.  Note that center 4, with a single patient, is represented by a single horizontal line at that meeting’s corresponding SDM score.

**
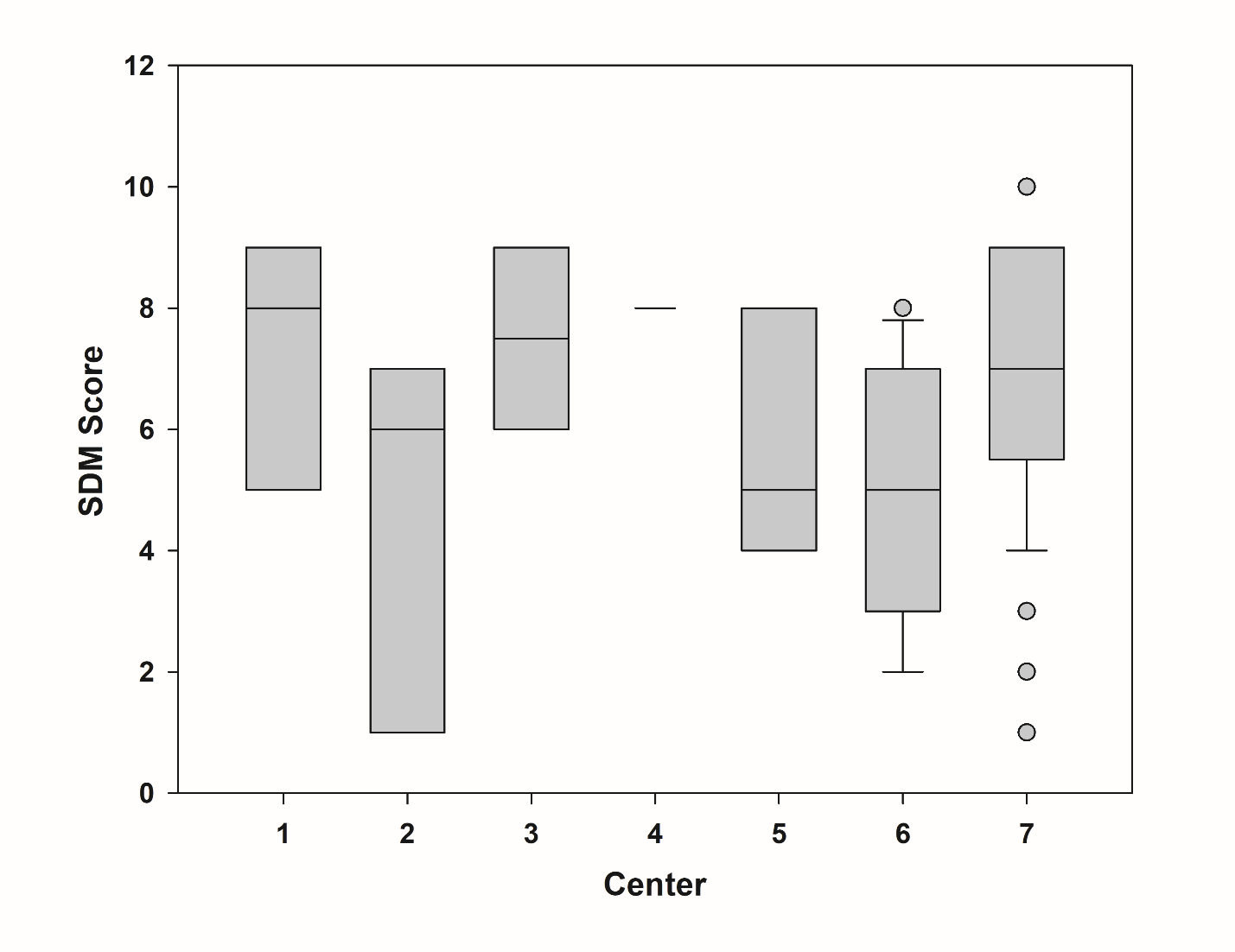
**

**Additional Figure 3:**

**Scatterplot of SDM score vs. survival discordance by center**

Each center is indicated by its number (a symbol 1 for center 1, symbol 2 for center 2, etc).


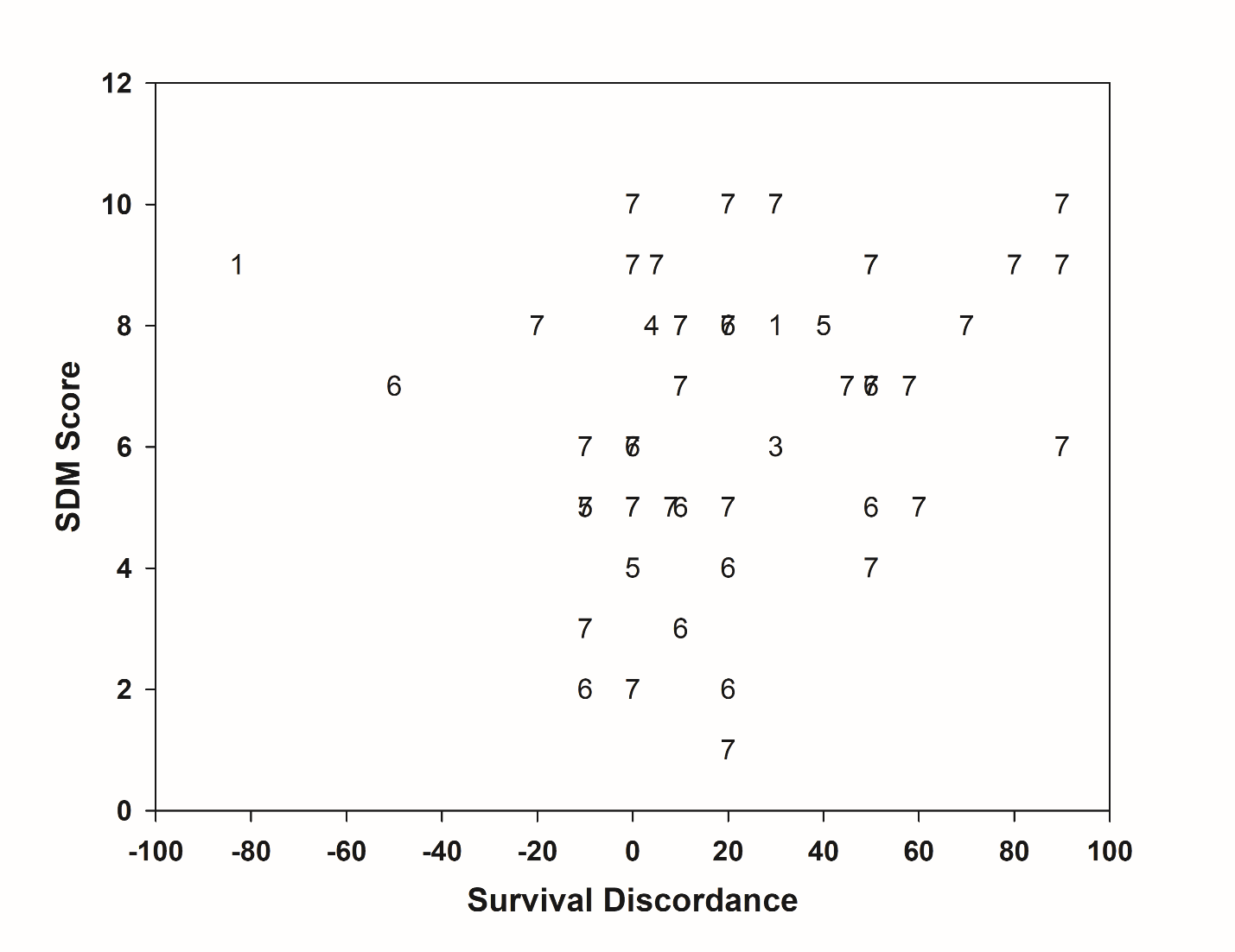


**Additional Figure 4:**

**Scatterplot of SDM score vs. survival discordance by center**

Given the small number of observations at all but 2 centers, we have also plotted SDM versus continuous survival discordance by cohort (cohort 1=centers 1 through 6, cohort 2=center 7). Cohort 1 is depicted by black dots, cohort 2 is shown as white dots. Although the stratified scatterplot suggests a possible difference in this association by cohort, the p-value for effect modification in a linear mixed model is 0.16.  Moreover, our models used dichotomized discordance (concordant vs. discordant) rather than continuous discordance score; the p-value for effect modification of the SDM versus dichotomized survival discordance is 0.78, suggesting negligible cohort-related differences in this association.


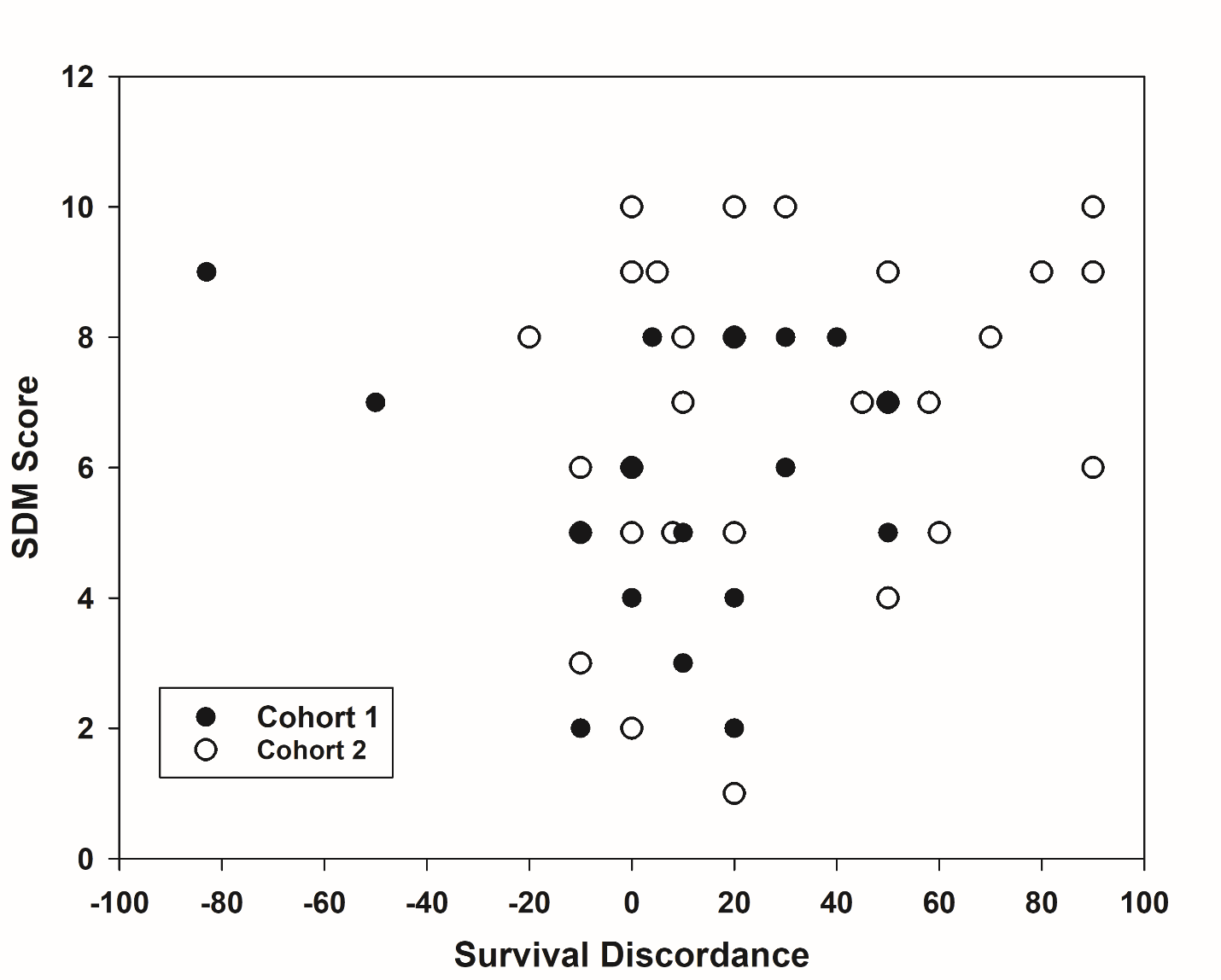

Supplement: Supplementary file 2 — Additional file 2. Additional details to the statistical analysis, SDM score subgroup analysis, Supplementray Table 2 and Figures S1–S4. [file 13054_2023_4693_MOESM2_ESM.docx]
